# *Ammi Visnaga* L., a Potential Medicinal Plant: A Review

**DOI:** 10.3390/molecules25020301

**Published:** 2020-01-12

**Authors:** Noha Khalil, Mokhtar Bishr, Samar Desouky, Osama Salama

**Affiliations:** 1 Faculty of Pharmaceutical Sciences and Pharmaceutical Industries, Future University in Egypt, Cairo 11835, Egypt; osalama@fue.edu.eg; 2 Arab Company for Pharmaceuticals and Medicinal Plants, (Mepaco-Medifood), El-Sharqiya 11361, Egypt; mbishr_2000@yahoo.com; 3 Faculty of Pharmacy, Minia University, Al Minya 61519, Egypt; dr_samar_yehia@yahoo.com

**Keywords:** *Ammi visnaga* L., γ-pyrones, khellin, visnagin, flavonoids, essential oil, muscle-relaxant

## Abstract

*Ammi visnaga* L. (*Visnaga daucoides* Gaertn., Family Apiaceae), also known as Khella Baldi or toothpick weed, is an annual or biennial herb indigenous to the Mediterranean region of North Africa, Asia, and Europe. The plant is known to have been used in traditional medicine a long time ago. Nowadays, it is used in modern medicine to treat many aliments such as renal colic and coronary insufficiency, and is used as an antioxidant, antifungal, and antibacterial, with a larvicidal effect on mosquito larvae. Peer-reviewed studies show that these pharmacological activities are due its valuable chemical constituents that include mainly essential oil, polyphenolic compounds including flavonoids, as well as γ-pyrones, represented mainly by khellin and visnagin. Its essential oil is reported to have antiviral, antibacterial, and larvicidal effects, while its flavonoid content is responsible for its antioxidant activity. Its γ-pyrone content has a powerful effect on facilitating the passage of kidney stones and relieving renal colic, in addition to having a relaxant effect on smooth muscle including that of the coronary arteries. The current review represents the progress in research on *A*. *visnaga* in terms of either its chemistry or biological activities. This review represents scientific support material for the use of the plant by the pharmaceutical industry.

## 1. Introduction

Since prehistoric times, plants have been employed in the treatment of many illnesses. Ancient humans used plants for the treatment of diseases by trial and error. They also noticed that animals avoid some plants while consuming others, and they followed their ways in dealing with nontoxic plants. Over time, people in various civilizations used plants in a planned and studied way. The Ancient Egyptians, Greeks, Romans, Chinese, and Indians had their own Planta Medica, which was a part of their *materia medica*. Many of the drugs mentioned in the Ebers Papyrus are still used today, including *Ammi visnaga* L., a plant that was known in ancient times as Pharaoh’s bread. Also, during the golden age of the Arabs, well-known physicians such as Avicenna, Al-Razy, Al-Antaki, and others mentioned many plants in their books, in an attempt to preserve, improve, and transmit the knowledge of ancient people [1]. This transferred knowledge is a corner stone for modern pharmacognosy and phytochemistry.

*Ammi visnaga* L. is a short annual or biennial herb indigenous to the Mediterranean region of North Africa, Asia, and Europe [2,3]. In Egypt, the plant is widely distributed in the Delta region, and surrounds the Nile River, particularly in Assiut and Minia governorates [4]. It is also widely cultivated by many people and companies aiming to use its extracts or active components in the pharmaceutical industry. It has been recently discovered and added to the flora of Croatia at altitudes of 380–460 meters above sea level [5]. The plant is also distributed throughout North America (North Carolina, Pennsylvania, Oregon, Alabama, California, Florida, and Texas), the Atlantic islands, Argentina, Mexico, and Chile. In Asia, the plant is found in Iraq, Iran, and other western and southern countries. The plant, especially its fruit, has a wide range of applications either in traditional or modern medicine. Despite the medical importance and applications of *A*. *visnaga* and its chemical constituents, no comprehensive literature review has been made for this plant. This review aims to report on the phytochemical constituents and pharmacological effects, as well as the applications of *A*. *visnaga* in the pharmaceutical industry. Data in this review was collected from 100 peer-reviewed articles, book chapters, and a WHO monograph (for the period 1947–2019) found in different online databases, such as PubMed, ScienceDirect, SciFinder, Google Scholar, and Scopus.

### 1.1. Synonyms 

Selinum visnaga E.H.L. Krause, Daucus visnaga L., Sium visnaga Stockes, Visnaga daucoides Gaertn., Ammi daucoides Gaertn., Ammi dilatatum St.-Lag., Apium visnaga L., Crantz, Carum visnaga L., Koso-Pol., Daucus gingidium L. ex DC [6].

### 1.2. Common Names

In Arabic countries *A*. *visnaga* has many common names such as Khella baladi, Khella, Khellah, Khellakl, Chellah, Kella, Gazar sheitani, Kammon habashi, Bizer Al-Khilla, Kulla, and Swak Al-Nabi. In Turkey, it is known as disotu, kilir, and hiltan.

In England, it is known as Pick tooth and Toothpick. In France it is known as Herbe aux cure-dents and in Germany as Zahnstocherkraut. The Berber calls it Tabellaout [6].

### 1.3. Morphology

*Ammi visnaga* is an herbaceous annual or sometimes biennial plant growing to a height of up to 1.3 meters and a diameter of the green aerial part of up to 1.2 meters. The root is a cylindrical tap root, and light brown in color. The root is straight or slightly tortuous, growing vertically. The surface of the root shows remnants of secondary fibrous rootlets. The main tap root measures up to 50 cm in length and 1.5 cm in the widest upper part. The stem is cylindrical, erect, highly branched, densely leafy, and glabrous. The alternate internodes are at a distance ranging from 5 to 8 cm in length, where light brown scaly leaves are present at the nodes. The stem measures up to 130 cm in length and 1.5 cm in diameter. Leaves are pinnate or ovate in shape with slender linear segments measuring about 20−30 mm in length and 0.5−1 mm in diameter. The margin of the segment is entire, while the apex is acute. Leaves are sessile in the upper shoots and have short petioles downwards. They are green at the upper region and greyish white in the lower part. Flowers form inflorescent white umbels, each of which is 6−10 cm across; flower stalks are elongated, up to 20 cm in length. With many bracts, the number of rays may be 50, 100, or 150 per umbel; each ray is slender and 2–5 cm long, unequally spreading when young. In the fruiting stage, rays become thick, rigid, and constricted on a discoid thickened base. They are surrounded with an involucre of long tripartite bracts. Bracteoles are numerous, 3–10 mm in length, entire, equaling flowers. Florets have a white corolla and calyx with minute inconspicuous sepals, and are 0.2 mm in length. The foliage part has a very characteristic smell and the flowers have their own special perfume. Fruits are ovoid oblong cremocarps, and brownish green with a violet tinge. They are laterally compressed with thick raised ridges and are glabrous. The umbels become dry and constricted on a discoid torus at the fruiting stage, measuring from 6 to 10 cm in length and 2 to 4 cm in width (at the widest part), and becoming light brown in color with characteristic odor and taste. The photosensitizing power of the plant sap must be taken into consideration, so it is advisable to handle the flowers or the whole plant with gloves or wash hands directly with water after handling [3,4,6].

### 1.4. Traditional Folk Medicinal Uses

The decoction and/or powdered plant has been traditionally used for the treatment of renal colic, mild anginal symptoms, treatment of abdominal cramps. It is also employed as a supportive treatment for mild obstruction of the respiratory tract in asthma or spastic bronchitis, and postoperative treatment of conditions associated with the presence of urinary calculi. The plant and its extracts are also popular in the treatment of vitiligo and psoriasis, and are used as a lithotriptic agent. It is generally used to dilate bronchial, urinary, and blood vessels without affecting blood pressure. It is also internally used as an emmenagogue to regulate menstruation, as a diuretic, and in the treatment of vertigo, diabetes, and kidney stones. An infusion of the aerial parts has also been used to treat headaches [2,7,8,9]. 

## 2. Chemical Review

The chemical constituents of *A*. *visnaga* are well known and have been reported by many researches in numerous studies throughout the years. Previous studies have reported on various chemical constituents in *A*. *visnaga*, including γ-pyrones, coumarins flavonoids, and essential oils. The quality and quantity of these secondary metabolites depend on the part of the plant analysed, as well as the growing conditions and the addition of any bioregulators [10].

### 2.1. γ-Pyrones and Coumarins

γ-Pyrones and coumarins are considered to be the major constituents of *A*. *visnaga*. They include:

γ-Pyrones (Furanochromone Derivatives)

Khellin and visnagin are the major ones [11,12,13,14,15], in addition to 4-norvisnagin, khellinol, visamminol, ammiol, and khellol [16]. Other important γ-pyrones include 5,7-dihydroxy-2-methyl-γ-pyrone-7*-O-*glucoside and pimolin (III) [17], as well as, khellinin, khellinone, and visnaginone [15].

Coumarins, which may be further divided into two sub-groups

Pyranocoumarins: represented by an angular-type dihydro-pyranocoumarin glucoside which was isolated from the fruits and named cis-khellactone-3′-β-d-glucopyranoside [18], in addition to visnadin [19], samidin, and dihydrosamidin [15].

Furanocoumarins such as xanthotoxin, ammoidin, bergapten, and psoralene are present, but only in small amounts [20].

**Figure 1:**
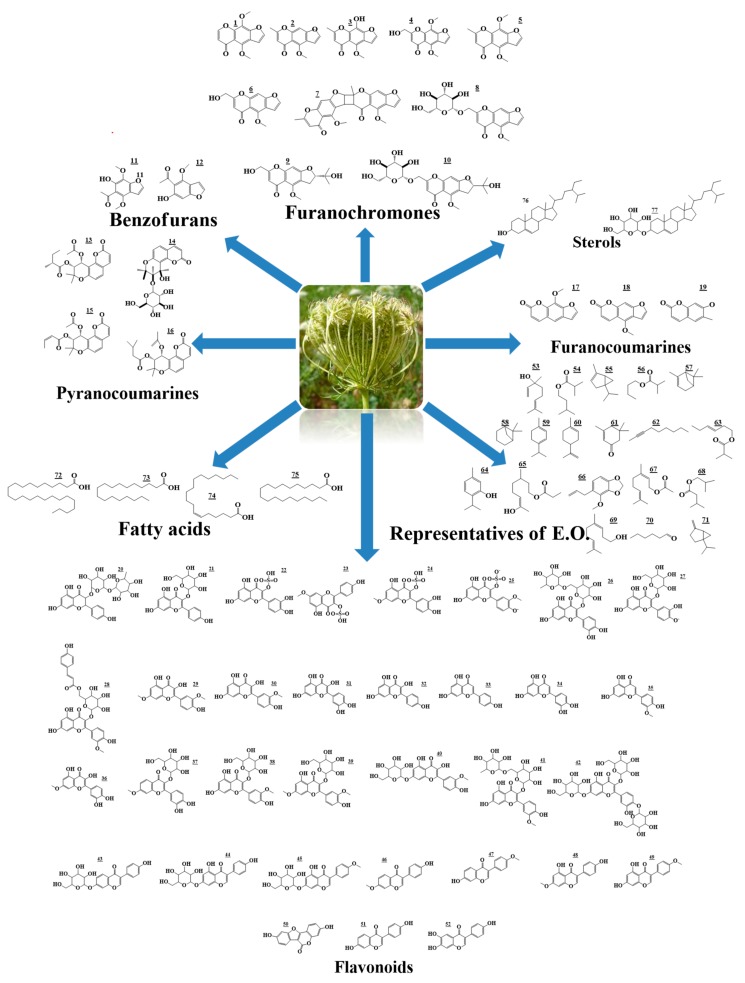
Metabolites previously isolated from *A*. *visnaga*.

The chemical structures of the aforementioned compounds have been determined using various spectroscopic techniques such as nuclear magnetic resonance, ultraviolet, mass spectroscopy, and infrared spectroscopy. The structures of previously identified γ-pyrones and coumarins are compiled in Table 1 and Figure 1.

### 2.2. Phenolic Compounds

Phenolic compounds are considered an important group of secondary metabolites that has been identified in *A*. *visnaga*, particularly in its aerial parts, by many researchers. They belong mainly to the flavonoids class. They are present in both aglycone and conjugated forms, and may be divided into:

#### 2.2.1. Flavonols

The main aglycones identified in *A*. *visnaga* include quercetin, kaempferol, rhamnocitrin, and rhamnetin [21], in addition to rhamnazin [16]. The flavonols in *A*. *visnaga* most commonly conjugate with either glucose or rutinose moeities. Flavonoidal glycosides include quercetin-3-glucoside, kaempferol-3-glucoside [21], and isorhamnetin 3-β-d-glucoside [17], as well as rhamnetin-3*-O-*glucoside, isorhamnetin-3*-O-*glucoside, rhamnazin-3-glucoside, isorhamnetin-7*-O-*glucoside, and quercetin-7,3,3’*-O-*triglucoside [22]. Those conjugated with rutinose comprise quercetin-3-rutinoside, kaempferol-3- rutinoside [21], and isorhamnetin-3*-O-*rutinoside [22]. *A*. *visnaga* is also considered a rich source of flavonoidal sulfates; The most commonly identified ones include quercetin 3-sulfate, rhamnocitrin 3-sulfate, rhamnetin, and isorhamnetin-3-sulfate [21].

#### 2.2.2. Isoflavones 

Represented by daidzin, genistin, sissotrin, isoformononetin, formononetin, prunetin, biochaninA, coumestrol, daidzein, and 6,7,4′-trihydroxyisoflavone. Their presence was identified by means of semipreparative HPLC with an enzyme-linked immunosorbent assay (HPLC-ELISA) along with high-performance liquid chromatography coupled with mass spectrometry (HPLC-MS) [23].

#### 2.2.3. Flavones as Apigenin, Luteolin and Chrysoeriol

The chemical structures of reported flavonoids are shown in Table 1 and Figure 1.

### 2.3. Essential Oil

*Ammi visnaga* L. was found to contain essential oils in different organs, but mainly in the umbels and fruits. Studies used either steam distillation or hydrodistillation to isolate the oil, and then analyse it by means of gas chromatography (GC) and gas chromatography coupled with mass spectrometery (GC-MS) to identify its components. The chemical constituents of the essential oils of *A*. *visnaga* are distributed mainly among the following groups: nonterpenes and monoterpenes [24], in addition to diterpenes and sesquiterpenes but only in very small amounts in the case of the latter two [25]. The most abundant constituents of *A*. *visnaga* essential oils were found to be isoamyl 2-methylbutyrate, isoamyl isobutyrate, iso-butyl-2-methylbutyrate, 2-methylbutyl 2-methylbutyrate, 2-methylbutyl isobutyrate, and isoamyl isovalerate [14,26]. A large group of the volatiles identified in *A*. *visnaga* belong to the monoterpenes class. They are either oxygenated or hydrocarbon monoterpenes, and the major components identified were linalool and thymol. They comprise the highest percentage among other monoterpenes. Other identified monoterpenes include α-thujene, α-pinène, β-pinene, and β-myrcene, but only in small amounts [27,28]. The nature of the main constituents of the essential oils of *A*. *visnaga* depend greatly on the plant part and its developmental stage. Many studies have dealt with the differences in the chemical composition of essential oils of *A*. *visnaga* and their respective amounts. They suggested that the differences could be associated with differences in biotypes and geographic origins along with variations in the environment such as soil type, solar radiation, and environmental stress. These factors could lead to the activation or inactivation of certain enzymatic groups, resulting in the up- or down-regulation of certain biosynthetic pathways [15]. The structures of the different constituents of the essential oils in *A*. *visnaga* are represented in Table 1 and Figure 1.

### 2.4. Sterols and Fatty Acids

Other classes of chemical compounds were found in various parts of *A*.*visnaga*; however, they are considered minor compounds and are found in low percentages in the plant. These include β-sitosterol and β-sitosterol-glucoside [29], in addition to palmitic, palmitoleic, stearic, petroselinic, linoleic, linolinic, arachidic, tetracosanoic acids, which were identified using high performance liquid chromatography-quadrupole time of flight (LC-QTOF) mass spectrometry [30].

**Table 1:** Secondary metabolites previously isolated from *A*. *visnaga*.

No.	Compound	Plant Origin	Plant Part	Reference
Furanochromones (γ-Pyrones)				
1	Khellin	Italy	Fruits	[11]
2	Visnagin			
3	Khellinol	Egypt	Fruits	[16]
4	Ammiol			
5	Visamminol			
6	Khellol			
7	Pimolin	Egypt	Fruits	[17]
8	Khellinin (khellol glycoside)	Turkey	Fruits	[13]
9	Cimifugin	Tunisia	Umbels	[10]
10	Prim***-O-***glucosyl cimifugin			
Benzofurans				
11	Khellinone	Egypt	Fruits	[16]
12	Visnaginone			
Pyranocoumarines				
13	Visnadin	Poland	Fruits	[19]
14	cis-khellactone-3′-β-d-glucopyranoside	Germany	Fruits	[18]
15	Samidin	Austria	Fruits	[31]
16	Dihydrosamidin			
Furanocoumarins				
17	Xanthotoxin	Egypt	Fruits	[32]
18	Bergapten			
19	Psoralen			
Flavonoids				
20	Kaempferol-3-rutinoside	England	Leaf, flower and fruit	[21]
21	Kaempferol-3-glucoside			
22	Quercetin3-sulfate			
23	Rhamnocitrin-3-sulfate			
24	Rhamnetin-3-sulfate			
25	Isorhamnetin-3-sulfate			
26	Quercetin-3***-O-***rutinoside	Algeria	Aerial parts	[22]
27	Quercetin-3***-O-***glucoside			
28	Isorhamnetin 3***-O-***β-d-glucoside	Egypt	Fruits	[17]
29	Rhamnazin	Egypt	Fruits and aerial parts	[16]
30	Isorhamnetin			
31	Quercetin	Iraq	Fruits and aerial parts	[33]
32	Kaempferol		Fruits	[33]
33	Apigenin			
34	Luteolin			
35	Chrysoeriol			
36	Rhamnetin	Algeria	Aerial parts	[22]
37	Rhamnetin-3***-O-***glucoside			
38	Isorhamnetin-3*****-O-*****glucoside			
39	Rhamnazin-3-glucoside			
40	Isorhamnetin-7***-O-***glucoside			
41	Isorhamnetin-3***-O-***rutinoside			
42	Quercetin 7,3,3′***-O-***triglucoside			
43	Daidzin	Czech Republic	Leaves and roots	[23]
44	Genistin			
45	Sissotrin			
46	Isoformononetin			
47	Formononetin			
48	Prunetin			
49	Biochanin A			
50	Coumestrol			
51	Daidzein			
52	4′,6,7-Trihydroxyiso-flavone			
Representatives of essential oil constituents				
53	Linalool	Tunisia	Fruits	[27]
54	Isoamyl-2-methylbutyrate			
55	α-Thujene			
56	Butyl isobutyrate			
57	α-Pinène	Tunisia	Leaves, stems, flower buds, roots, umbels and fruits	[24,27]
58	β-Pinene			
59	α-Terpinene			
60	Limonene			
61	α-Isophorone	Algeria	Fresh aerial parts	[26]
62	2-Nonyne			
63	Hexenyl isobutanoate			
64	Thymol			
65	Citronellyl propionate			
66	Croweacin			
67	Geranyl acetate			
68	Isobutyl isovalerate	Algeria	Fruits and fresh aerial parts	[34]
69	Nerol		Fresh aerial parts	
70	Hexanal	Algeria	Umbels	[28]
71	Sabinene			
Fatty acids				
72	Tetracosanoic acid	Egypt	Aerial parts	[29]
73	Stearic acid			
74	Petroselinic acid	Egypt	Fruits	[30]
75	Arachidic acid			
Sterols				
76	β-sitosterol	Egypt	Aerial parts	[29]

## 3. Pharmacological Review

### 3.1. Kidney Diseases

*Ammi visnaga* L. has been used in folk medicine by the Middle Eastern population since ancient times. The fruit decoction was used for the treatment of renal colic by the ancient Egyptians [35], as a treatment of kidney inflammation in Iraq [36] and Palestine [37], and in the treatment of urolithiasis and prostatic pain in Algeria [38]. Its use had spread to the extent that it was regarded as the most recommended species for the treatment of urinary tract infections [39]. Several studies have focused on the diuretic activity of *A*. *visnaga*; it has been shown to be effective in the treatment of nephrolithiasis and uremia [40]. Its use in the treatment of kidney disorders is commonly coupled with khellin and visnagin, i.e., the major γ-pyrones of *A*. *visnaga*. They have been shown to protect the renal epithelial cell damage from oxalate and calcium oxalate monohydrate crystals, and to prevent the oxalate formation that is associated with hyperoxaluria by increasing the urinary pH and citrate concentration, along with a decrease of urinary oxalates [41,42,43,44,45]. The pleiotropic effects of khellin and visnagin on urolithiasis have been intensively studied by many researchers [46,47,48]. Bhagavathula reported that a patient suffering from recurrent urethral stones showed complete recovery after treatment with *A*.*visnaga* fruit for ten days [49]. Recently, an experiment was conducted in an approach aiming to further explain the mode of action of *A*. *visnaga* in inhibiting the nucleation and preventing the crystallization of kidney stone [50].

### 3.2. Antispasmodic and Vasodilating Effects

The vasodilating properties of *A*. *visnaga* have been investigated by several researches. It has been established as a bronchodilator and coronary medication in the treatment of angina pectoris due to its peripheral and coronary vasodilator activity [51], in addition to being an antiasthmatic and a vasodilator, as well as an effective muscle relaxant agent without affecting blood pressure [3,52]. The vasodilating properties of *A*. *visnaga* are associated with its two major γ-pyrones, khellin and visnagin, along with the pyranocoumarin, visnadin. Both khellin and visnadin have been proven to possess calcium antagonistic activity, which, in turn, yields vasodilating activities. Visnadin has been shown to possess both peripheral and coronary vasodilator activities, and is thus used for the treatment of angina pectoris. It preferentially inhibits the contractile responses mediated by Ca^2+^ entry through L-type Ca^2+^ channels, and at high concentrations, it may also interfere with other sites involved in vascular smooth muscle contraction [53,54,55,56,57,58]. The vasodilating effect of visnagin is a result of inhibiting the vascular smooth muscle contractility at multiple sites, and weakly inhibiting the hydrolytic activity of the cyclic nucleotide phosphodiesterase (PDE) isozymes [59,60,61].

### 3.3. Antidiabetic Activities

The use of *A*. *visnaga* as an antidiabetic agent is considered famous in many cultures, such as those of Palestine, Moroco, and the Sefrou region [35,62,63,64]. An aqueous extract of *A*. *visnaga* was shown to possess a significant hypoglycemic effect when given to both normal and streptozotocin diabetic rats [65]. additionally, a decoction prepared from the fruits of the *A*.*visnaga* had the ability to reduce blood glucose levesl by 51% in normoglycemic rats, compared to an oral hypoglycaemic agent (tolbutamide) [66]. 

### 3.4. Treatment of Vitiligo

Since 1982, khellin has been shown to be effective in both oral and topical photochemotherapy for the treatment of vitiligo. A study done by Orecchia indicated that treatment with a gel formulation of khellin based upon a water/2-propanolpropylene glycol (khellin-WPG) system combined with ultraviolet A (UVA) significantly improved the clinical outcome of patients with vitiligo by facilitating the availability of the drug in the skin [67]. The treatment was proven to be safe for both short- and long-term treatments [68]. Later on, an open clinical trial was conducted to investigate the efficacy and safety of treatment with khellin encapsulated in L-phenylalanine stabilized phosphatidylcholine liposomes in combination with ultraviolet A/ultraviolet B (UVA/UVB) light therapy (KPLUV) in 74 patients suffering from vitiligo. The treatment was shown to be highly effective, and did not cause any side effects [69]. Furthermore, the melanin biosynthesis inhibitory effects of khellin were investigated using a B16 melanoma cell line, and showed a potent inhibitory activity compared to arbutin, which was used as a positive control in the experiment [29]. Moreover, the additional value of combining blister roof transplantation (BRT) with khellin in liposomes and ultraviolet light (KLUV) in the treatment of recalcitrant vitiligo patches has been investigated; the results showed that almost 75% of the treated patients were extremely satisfied with the result [70].

### 3.5. Anti-inflammatory Effect

The anti-inflammatory effects of *A*. *visnaga* have been investigated, and it was shown that, depending on its visnagin content, it caused a decrease in mRNA expression and the release of TNF-α, IL-1β, and IFNγ. In addition, visnagin reduced LPS-induced IL-6 and MCP-1 mRNA level, thus suggesting that the anti-inflammatory effect of visnagin may be due to the inhibition of transcription factors such as AP-1 and NF-κB [71]. Moreover, Kwon et al. suggested that visnagin had a neuroprotective effect in terms of suppressing kainic acid-induced pathogenesis in the brain, and that these neuroprotective effects are associated with its anti-inflammatory effects [72]. 

### 3.6. Antimicrobial Effect

Several studies have reported on the antimicrobial effects of the different extracts of *A*.*visnaga* The alkaloidal and sesquiterpene lactone fractions have shown activity against *Candida* species [73], while the ethanolic extracts of fruits showed a significant inhibition of the growth of *Mycobacterium tuberculosis* [74]. In addition, the fruit’s aqueous extract inhibited the growth and aflatoxin production of *Aspergillus flavus* in a dose-dependent manner [75]. Additionally, remarkable activity was revealed for the aqueous and hydroalcoholic stem extracts of *A*. *visnaga* against *Streptococcus mutans*, *Streptococcus salivarius*, and *Streptococcus sanguis* [76]. In an approach aimed to evaluate the possibility of using *A*. *visnaga* extracts in pharmaceutical and food preservation systems, a study showed that the fruit ethanolic extract was the most active extract against the Gram-positive bacteria *Enterococcus faecalis*. Moreover, the same extract revealed antimicrobial activity against the Gram-negative bacteria *Escherichia coli* and *Klebsiella pneumoniae* [77]. Several studies have focused on the antimicrobial effects of the essential oils of the *A*. *visnaga* L., showing their effectiveness against various microorganisms such as *Escherichia coli*, *Pseudomonas aeruginosa*, and *Klebsiella pneumoniae* strains [25,26,78,79]; however, they showed weak antifungal activities [34]. Razzaghi-Abyaneh identified components that strongly inhibited aflatoxin formation in toxigenic fungi, e.g., khellin, xanthotoxin, and bergapten [20].

### 3.7. Cytotoxic Activity

In recent years, focus has been directed to the discovery of new cytotoxic agents, and attempts have been made to investigate the cytotoxic activities of many medicinal plants, including *A*. *visnaga*. In 2004, khellin was isolated, and its cytotoxicity was evaluated against four human tumour cell lines: HT-29 (colorectal cancer), MCF-7 (breast cancer), HEp-2 (larynx cancer), and MKN-45 (gastric cancer). However, the results were not promising, and the substance did not show significant cytotoxic activity at the tested concentrations against the four cell lines [80]. On the other hand, khellin showed mild to moderate activity when tested against the hepatocarcinoma cell line (HepG2) [81]. An ethanolic extract of *A*. *visnaga* also showed inhibitory effects on both Hela (cervical cancer) and MCF7 cell lines [82]. The cytotoxic activity of isolated khellin and visnagin against four human cell lines, Hela (cervical carcinoma), Hep-G2 (liver carcinoama), HCT 116 (colon carcinoma), and MCF7 (breast carcinoma), was further investigated; the results revealed good cytotoxic activity of both γ-pyrones against the Hep-G2 cell line [83]. 

### 3.8. Antioxidant Activity

Very few studies have examined the antioxidant properties of *A*. *visnaga*. The free radical scavenging activity of the butanol extracts of the aerial parts of *A*. *visnaga* has been investigated, showing equivalent antioxidant activity, i.e., an IC_50_ equals to 8.77 ± 0.2 µg/mL, to the standard antioxidant rutin (IC_50_ = 3.01 ± 0.2 µg/mL) [22]. Another study examined the antioxidant activity of essential oils isolated from the umbels of *A*. *visnaga*; however, the results showed only very weak activity [28].

### 3.9. Hair Loss 

The topical application of *A*. *visnaga* for hair loss has been studied. A lotion for hair scalp composed of visnadin and other constituents led to an increase in arterial and arteriolar sphygmic activity in the subpapillary plexus, leading to an improvement in local microcirculatory flow [84]. 

### 3.10. Antimutagenic Effect

In a study aiming to evaluate the antimutagenicity spectrum of *A*. *visnaga*, khellin showed inhibition to mutagenicity of promutagens benzo[a]pyrene, 2-aminofluorene, and 2-aminoanthracene in *Salmonella typhimurium* T98, while visnagin showed higher toxic activity. Meanwhile, the total extract from *A*. *visnaga* fruit showed higher inhibition potency than khellin alone against 2-aminoanthracene, 1-nitropyrene, and daunomycin. This was attributed to the presence of additional inhibitors such as coumarins, or to the synergistic effects with the accompanying compounds [85]. 

### 3.11. Cardiovascular Activity

It is well known that *A*. *visnaga* extract or its active principals exert a relaxant effect on smooth muscles, even those of coronary arteries. It was found that intravenous injection of visnagin lowered the blood pressure with no change in the heart rate. It was also found that samidin and khellol glycoside induced a positive inotropic effect on the heart, while visnadin in a concentration 60 µg/mL increased coronary blood flow in isolated guinea pig heart. On the other hand, it was found that khellin, samidin, dihydrosamidin, and visnadin effectively normalized the electrocardiogram of ischemic myocardia in a dog. It seems that khella extracts or active principals improve the blood supply to coronary smooth muscles, where it dilates the coronary arteries without affecting the heart rate. The administration of khellin by oral or intramuscular injection gave good results in treating angina pectoris, and is favored in case of coronary thrombosis. As a result, khellin in a concentration of 50 mg/mL can help prevent angina pectoris with no side effects, although it is weaker than glyceryl trinitrate [54,59,60,61].

### 3.12. Immunostimulatory Activity

*A*. *visnaga* total and protein extracts were found to have immunostimulatory effects. Extracts were tested using an MTT (3-(4,5-dimethylthiazol-2yl)-2,5-diphenyltetrazolium bromide) assay on splenocytes with or without stimulation by concanavalin-A (Con-A), a mitogenic agent used as a positive control. This could explain the traditional use of such a plant [86].

### 3.13. Other Reported Activities on Human

Gouda obtained results that suggested that *A*. *visnaga*, among other plants, might have analgesic activity [87]. Also, Bhagavathula et al. suggested that *A*. *visnaga* fruits have a significant effect on increasing HDL-cholesterol levels, highlighting the hypothesis that it could be used in treating hypertriglyceridemia [49]. 

### 3.14. Larvicidal and Insecticidal Activities

Many natural products isolated from plants could be used as alternative treatments with larvicidal and insecticidal activities. For this purpose, the larvicidal and insecticidal properties of *A*. *visnaga* were studied, and the toxicity of khellin was investigated against nymphs *Oncopeltus fasciatus* (Hemiptera) and the larvae of *Aedes aegypti* (Diptera), where it showed great activity [88]. Studying the acaricidal and ovicidal activity of khellin and visnagin against *Tetranychus urticae* showed that both khellin and visnagin were highly promising, and could be used for the development of new botanical acaricides from *A*. *visnaga* [89], as well as that they were both phytotoxic to model species lettuce (*Lactuca sativa*) and duckweed (*Lemna paucicostata*) [90]. The fruit extract was found to possess an inhibitory action on the lipid content in haemolymph of nymphs and adults [91], while the *n*-butanolic extract of *A*.*visnaga* was also shown to prevent the activity of Glutamic oxaloacetic transaminase (GOT) and Glutamic pyruvic transaminase (GPT) in haemolymph and fat bodies of last instar nymphs and newly-emerged adult females of the dangerous desert locust, *Schistocerca gregaria* [92].

### 3.15. Herbicidal Activity

A study done in Argentina found that the dichloromethane extract of *A*. *visnaga* had a significant herbicidal effect. Phytotoxicity fractionation was done, and the fraction that contained khellin and visnagin was found to be responsible for its significant herbicidal activity [90].

## 4. Standardization of *A*. *visnaga* Fruit

Several pharmacognostic and phytochemical parameters for the standardization of *A*.*visnaga* fruit have been mentioned in the WHO monograph, among which the most important is that the fruit should contain not less than 1% γ-pyrones calculated as khellin, as determined using spectrophotometry [6].

## 5. Methods for Analysis of γ-Pyrones (Khellin and Visnagin)

Several methods have been applied for the determination of the γ-Pyrones percentage in *A*. *visnaga* (i.e., in the root, aerial parts, umbel, and fruit) extracts or pharmaceutical products. These methods include the spectrophotometric method, the colorimetric method, GC, and HPLC:

### 5.1. Spectrophotometric Method 

This depends on the chromatographic adsorption of the impurities from the alcoholic extract, and then determining the extinction coefficient in the region of 220−350 nm. A calibration curve for standard Khellin was prepared for such a quantitative determination [93].

### 5.2. Gas Chromatography

This simple, fast, and precise method was applied using a capillary column DB-17 and injecting a chloroform extract of khellin with an internal standard [94].

### 5.3. Capillary Electrophoresis

Capillary electrophoresis has been employed for the analysis of *A*. *visnaga* extracts. Capillary electrophoresis is considered a useful, simple, and rapid technique for the identification and determination of khellin and visnagin in *A*. *visnaga* fruits. This method needs only a minute sample without any preseparation process. The process is time and reagent efficient. In order to separate the nonionic khellin in capillary electrophoresis, an interaction with a charged carrier in the buffer should be provided by including a surfactant in the mobile phase [95]. 

### 5.4. HPLC Methods

Several validated HPLC methods have been applied for the separation and simultaneous quantitation of different γ-pyrones. A calibration curve is prepared and validated methods are applied using reversed phase technique. These methods are selective, time saving, and widely applicable in quality control for the herb, its extracts, or pharmaceutical products [96,97,98,99]. 

### 5.5. Supercritical Carbon Dioxide Fluid Chromatography

Supercritical fluid chromatography (SFC) is a separation technique which uses instrumentation that is almost identical to that used in HPLC. Complex mixtures can be separated and the amount, and sometimes the identity, of the individual components in the mixture can be determined. A solution of the sample is injected into a high-pressure flow stream that sweeps the sample into a tube or column filled with fine particles. The individual components in the sample interact differently with the surface of the particles, and are separated in time and space as they pass through the column. The components emerge from the column at different times, as Gaussian or pseudo-Gaussian peaks, and pass through a detector. As a rule of thumb, any compound that is soluble in methanol or a less polar solvent is a good candidate for separation by SFC; therefore, it is very suitable for the determination of closely related structures and complex mixtures of γ-Pyrones in *A*. *visnaga* [100]

## 6. Pharmaceutical Products 

Worldwide, many pharmaceutical products contain *A*. *visnaga* extract or active principals. The following are some examples.

Egypt

- Kellagon® (capsules and effervescent sachets), produced by Arab Company for Pharmaceuticals and Medicinal Plants (Mepaco).
- Khellalgin® ampoules, produced by Misr Company.
- Glucolynamine®, produced by Memphis Company.

Germany

- Aesrutal® S tablets, produced by Steigerwald Company.
- Carduben® capsules, produced by Madaus Company.
- Khellangan N® tablets, produced by Ardeypharm Company.
- Steno-logs N® tablets, produced by Loges Company.
- Dr. Reckweg R66 arrythmin liquid, produced by Reckweg&Co.

France

- *Ammi visnaga* drops, produced by Biotron Laboratories.
- *Ammi visnaga* granules, produced by Biotron Laboratories.
- *Ammi visnaga* globules, produced by Biotron Laboratories.

Spain

- Vibeline®, produced by Promesa Company.
- *United States of America*
- *Ammi visnaga* Pellets, produced by Washington Homeopathic products.
- CL-N liquid, produced by Vitality Works, Inc.
- CN-Tone liquid, produced by Apotheca Company.
- Dr. Reckweg R66 arrythmin liquid, distrubed by Dr. Reckweg America Inc.
- GB-TONE liquid, produced by Apotheca Company.
- Liver Tonic II liquid, produced by Apotheca Company.

Romania

- Kellagon® capsules, produced by Mepaco Egypt and distributed by Sprint Pharma Company.

## 7. Dosage, Contraindications and Warnings

The daily recommended dose from *A*. *visnaga* fruit ranges from 0.05 to 0.15 g. For other dosage forms, the dose must be mentioned in the insert leaflet, as directed by the physician. It is expected to be the equivalent to the aforementioned dose of the fruit. Nothing has been reported regarding contraindications, except that such treatments must be avoided during pregnancy. Also, there are no warnings regarding the dried drug or its preparations, but it is advisable that patients receiving Khella treatment are not exposed to direct sun light. Care must be taken while handling the fresh plant to avoid the photosensitizing effect of the sap which is present in various organs.

## 8. Conclusions

*Ammi visnaga* has been used in traditional medicine for millennia. At present, it is widely cultivated by many peoples and companies aiming to use its extracts or active principals in the pharmaceutical industry. It is used in modern medicine to treat many aliments such as renal colic and coronary insufficiency, and as an antioxidant, antifungal, and an antibacterial with a larvicidal effect on mosquito larvae. The current review aimed to report on the main phytochemical constituents and pharmacological effects, as well as applications of *A*. *visnaga* in the pharmaceutical industry. The literature reviewed in this study recommends new research on and applications of this plant, especially in the field of the reliable production of herbal medicine.
